# Most UK scientists who publish extremely highly-cited papers do not secure funding from major public and charity funders: A descriptive analysis

**DOI:** 10.1371/journal.pone.0211460

**Published:** 2019-02-27

**Authors:** Charitini Stavropoulou, Melek Somai, John P. A. Ioannidis

**Affiliations:** 1 School of Health Sciences, City, University of London, London, United Kingdom; 2 Faculty of Medicine, School of Public Health, Imperial College London, London, United Kingdom; 3 Stanford Prevention Research Center, Stanford University School of Medicine, Stanford, CA, United States of America; 4 Department of Health Research and Policy, Stanford University School of Medicine, Stanford, CA, United States of America; 5 Department of Statistics, Stanford University School of Humanities and Science, Stanford, CA, United States of America; 6 Meta-Research Innovation Center at Stanford (METRICS), Stanford University, Stanford, CA, United States of America; Indiana University Bloomington, UNITED STATES

## Abstract

The UK is one of the largest funders of health research in the world, but little is known about how health funding is spent. Our study explores whether major UK public and charitable health research funders support the research of UK-based scientists producing the most highly-cited research. To address this question, we searched for UK-based authors of peer-reviewed papers that were published between January 2006 and February 2018 and received over 1000 citations in Scopus. We explored whether these authors have held a grant from the National Institute for Health Research (NIHR), the Medical Research Council (MRC) and the Wellcome Trust and compared the results with UK-based researchers who serve currently on the boards of these bodies. From the 1,370 papers relevant to medical, biomedical, life and health sciences with more than 1000 citations in the period examined, we identified 223 individuals from a UK institution at the time of publication who were either first/last or single authors. Of those, 164 are still in UK academic institutions, while 59 are not currently in UK academia (have left the country, are retired, or work in other sectors). Of the 164 individuals, only 59 (36%; 95% CI: 29–43%) currently hold an active grant from one of the three funders. Only 79 (48%; 95% CI: 41–56%) have held an active grant from any of the three funders between 2006–2017. Conversely, 457 of the 664 board members of MRC, Wellcome Trust, and NIHR (69%; 95% CI: 65–72%) have held an active grant in the same period by any of these funders. Only 7 out of 655 board members (1.1%) were first, last or single authors of an extremely highly-cited paper. There are many reasons why the majority of the most influential UK authors do not hold a grant from the country’s major public and charitable funding bodies. Nevertheless, the results are worrisome and subscribe to similar patterns shown in the US. We discuss possible implications and suggest ways forward.

## Background

The UK is home to some of the largest public and charity funding bodies of health research in the world.[1] In 2014, the UK spent approximately £3bn on health research through public and charity sources.[2] Although the extent to which this public investment reaches its full potential is not known–and indeed in the past has been questioned [3]- a number of cases have demonstrated that improvements in public health, discovery of new treatments and the implementation of more efficient ways to deliver services in the NHS would not have been possible without this financial support.[4]

Yet, there is very little published evidence on who are the recipients of these grants and whether public and charity funders in the UK award the most influential researchers. In the US, a previous analysis observed that the majority of the most widely cited US authors do not hold funding as Principal Investigators (PIs) from the National Institutes of Health (NIH), and what is more, NIH funding seems to recycle similar ideas rather than promoting new ones.[5] The same analysis found that the members of the study sections who reviewed grant proposals did not have particularly high citations. Citations have many limitations and they are not the only criterion indicating academic excellence [6] or translational potential. But when papers are extremely highly-cited, it does at a minimum signal that the paper has had a wide impact in the discourse of the scientific community through other articles.

In this paper, we explore the link between extremely highly-cited papers and public and charitable funding in the UK. Following an analogous methodology to the previous US evaluation,[5] we searched the Scopus database to identify authors from the UK with extremely highly-cited papers, which are defined as papers with more than 1,000 citations. We searched for papers that were published from 2006, the year the National Institute for Health Research (NIHR) was established. We then examined whether these authors have held funding as PIs from the country’s largest public and charity funders. Finally, we identified the members from the board of these funders to explore whether they have received a grant as PIs during the same period and whether they have authored any of the extremely highly-cited papers.

Our findings suggest than only a minority of the UK-based researchers who publish the most influential papers receives funding from the UK’s major public and charity bodies. This raises questions about how appropriate funding allocation mechanisms are in supporting highly-cited research in the UK.

## Methods

### Scopus analysis

To identify the most highly cited papers and their UK authors, we used the Scopus online database (https://www.elsevier.com/solutions/scopus). We searched for papers published in English from January 2006 till February 2018, focusing on journal articles in health-related fields. In particular we used the Scopus subject codes for Biochemisty, Genetics and Molecular Biology (BIOC), Dentistry (DENT), Health Professions (HEAL), Immunology and Microbiology (IMMU), Medicine (MEDI), Neuroscience (NEUR), Nursing (NURS) and Pharmacology, Toxicology and Pharmaceutical (PHAR). We narrowed down our search to UK only, using the country filter in the Scopus interface, which means that only articles with at least one UK author would be included. The corresponding Scopus search string can be found in the Appendix.

One of the authors (CS) sorted the papers by the number of citations and downloaded the ones that have been cited more than 1000 times. Nicholson and Ioannidis [5] had used the same threshold in the US, therefore by following their definition, we can compare our findings with the only other study that looks at funding of authors of extremely highly-cited papers. Moreover, gaming (e.g. self-citations and cross-citation from authors of the same team) may produce spuriously highly-cited papers, but this is most difficult to achieve when the threshold is extremely high. From the online browser, we downloaded our search results in CSV files. We focused on the main authors of each paper, which means first, last and single authors of each extremely highly cited paper and we identified their affiliation from Scopus. We then searched the authors’ institutions websites, Google and LinkedIn profiles to identify the current affiliation of these authors. This was performed to check whether these individuals were still in academia, whether they had moved out of the UK or whether they had retired.

### Funding bodies

In the UK, public funding for health research (medical, biomedical, life science, and other health-related fields) is provided via two main channels: Research Councils or the Government’s various Departments. We chose to analyse data from the largest of each category: the Medical Research Council (MRC) and the Department of Health’s NIHR, respectively. We also included in our analysis the Wellcome Trust, the UK’s biggest charity funding body. The three funders together—the MRC, the NIHR and the Wellcome Trust- account for 66% of the total £3b spent on health relevant research in 2014 in the UK.[7] In addition, their priorities are rather complementary. UK charities provide nearly half of all funding in aetiology, detection and treatment development; Research Councils fund underpinning research; while the Department of Health focuses mostly on treatment evaluation, disease management and health services research.[7]

### Principal investigators

For each one of the extremely highly cited authors, we searched the MRC, the Wellcome Trust and the NIHR databases of funded projects to see how many of them have held a grant as PI. Whenever an author was the director of an MRC or BRC/NIHR centre, they would be classified as leaders of a funded project.

We searched the databases of the NIHR to identify all PIs of grants awarded between 2006, the year the NIHR was launched. We looked at all of NIHR’s nine main programmes, namely Efficacy and Mechanisms Evaluation (EME); Health Services and Delivery Research (HS&DR); Health Technology Assessment (HTA); Research for Patient Benefit (RfPB); Invention for Innovation (i4i); Public Health Research (PHR); Policy Research Programme (PRP); Programme Grants for Applied Research (PGfAR); Systematic Reviews (SR). For four of these programmes (RfPB, i4i, PGfAR, PRP), the NIHR provides a detailed list of all the grant holders on its website (https://www.nihr.ac.uk/funding-and-support/funding-for-research-studies/funding-programmes/). The last time the lists were updated was December 2017. All other programmes have their projects listed on the NIHR Journals Library’s website (www.journalslibrary.nihr.ac.uk).

The MRC has a list of all its funded projects on its website (https://www.mrc.ac.uk/research/funded-research/), which, at the time of analysis (February 2018), included all the projects funded by the council till 2^nd^ November 2017. Similarly, a list of all the grants awarded by the Wellcome Trust between 1 October 2000 and 30 September 2017 is publicly available on the Trust’s website (https://wellcome.ac.uk/funding/managing-grant/grants-awarded#grantholders).

### Board members

We then focused on the current board members of the MRC, the Wellcome Trust and the NIHR (as of February 2018), to identify how many of these members have held a grant from the body they serve for or from any of the other two. All their members appear on the funder’s website.

We excluded from our analysis members who do not work in the UK, those who are members of the general public and those who work for non-academic institutions, to make this group comparable to the highly-cited authors group. We also excluded non-voting members and advisory panels as these provide advice on funding priorities but do not decide on who gets awarded the grants.

We included in our analysis 188 eligible board members of the MRC from 12 different programmes and 168 members of the Wellcome Trust from 21 programmes. We also analysed 317 members from eight of the NIHR’s programmes. We excluded the NIHR’s i4i programme, as most of its members are from the industry.

For each of the eligible board members, one of the authors (MS) searched the Scopus database to identify their paper with the highest citation count between January 2006 and 23^rd^ April 2018 (last update). We examined papers where they were the first, last or single author.

## Results

### Extremely highly-cited papers and their authors in scopus

Our query of the Scopus database yielded a total of 1,370 papers in health and related fields had received more than 1,000 citations between January 2006 and February 2018. When limiting the results to UK affiliations, using the Scopus affiliation filter, 333 out of these 1,370 papers had at least one author from a UK institution, though not necessarily one of the main ones. Overall, 110 of these studies were classified as basic science (33%), 88 were randomized controlled trials (26.4%), 50 were epidemiological studies (15%), 32 were systematic reviews and meta-analyses (9.6%), 24 were guidelines and recommendations (7%), 17 involved classifications, definitions and diagnosis criteria (5%) and 12 did not fall into any of the above categories (3%).

For each one of these 333 studies, we identified the first, last or single author’s affiliation and selected those authors who were associated with a UK institution at the time the paper was published. We included in the analysis authors with multiple affiliations as long as one of the affiliations was based in the UK. This resulted in a subset of 175 papers and a total of 223 authors with a UK affiliation who were first, last or single authors of a paper with more than 1000 citations published between 2006 and February 2017. The 175 papers included 76 basic science articles (43.4%), 33 randomized controlled trials (18.9%), 27 epidemiological studies (15.4%), 21 systematic reviews and meta-analyses (12%), 7 guidelines and recommendations (4%), 5 classifications, definitions and diagnosis criteria (2.9%) and 6 other types of papers (3.4%).

**Fig 1:**
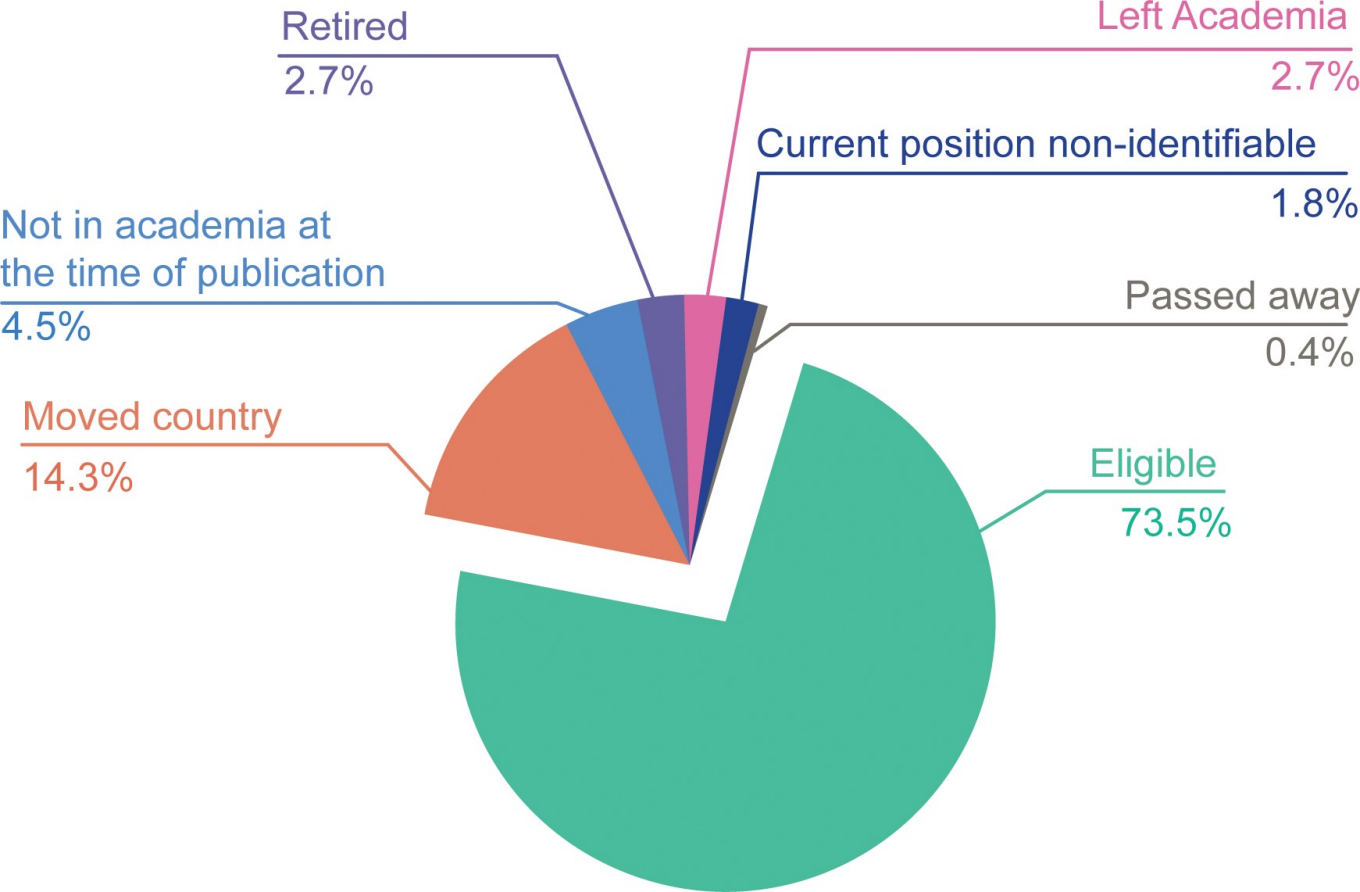
UK authors of extremely highly-cited papers who are eligible or not to apply for a grant.

Of those 223 authors, only 43 (19%) were women, which is in line with gender disparities observed often in science.[8] Ten authors (4.5%) were not in academia at the time of the highly cited publication. Seven of them worked for a pharmaceutical company and three in other non-academic institutions. These authors were still not in academia as of February 2018, and we have therefore decided to exclude them from further analysis as they are not eligible to apply for a grant from one of the three funding bodies included in our study. We also excluded from the analysis 32 authors (14.3%), who had left the UK and another 6 (2.7%), who worked in an academic institution at the time of publication but had left academia by February 2018. Of those 38 authors that we excluded, only 7 (18%) had received funding from one of the three funding bodies before leaving the UK or academia. In addition, at the time of analysis 6 individuals (2.7%) had retired, 1 (0.4%) had passed away and 4 (1.8%) had left their latest listed institution but could not be definitely tracked down as retired and/or deceased (Fig 1). These individuals were also excluded from our analysis.

After excluding non-eligible authors, we were left with a list of 164 individuals from the UK who had published a paper with more than 1000 citations and were eligible to apply as PIs for a grant from these 3 UK funding bodies.

**Fig 2:**
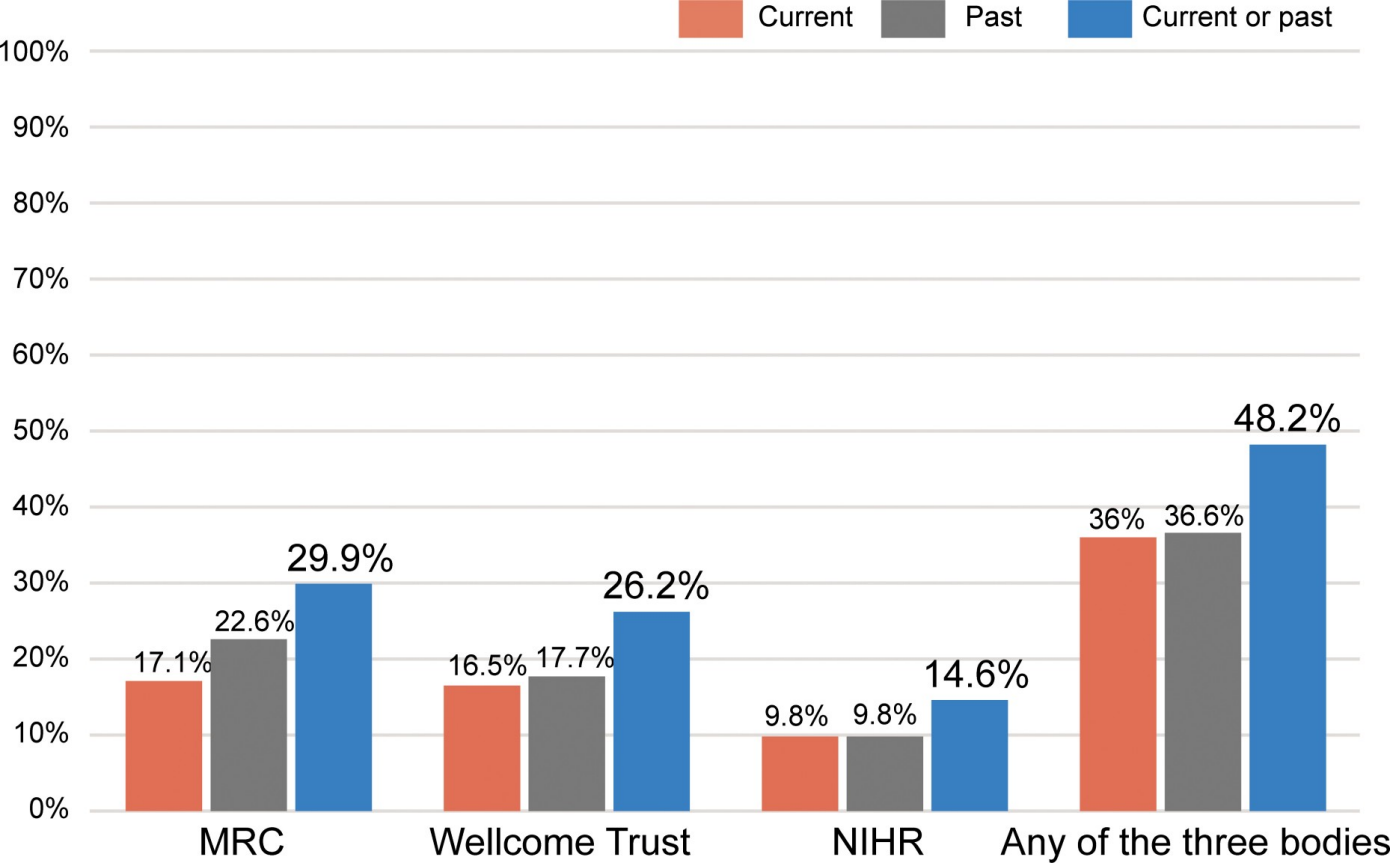
Percentage of authors of extremely highly-cited papers who have been principal investigators in a grant.

Out of these 164 individuals, we found that 59 (36.0%; 95% CI: 28.6–43.3%) currently hold an active grant as PIs from one of the three major funders, while 60 (36.6%; 95% CI: 29.2–44.0%) did so in the past (Fig 2). That brings the total number of authors of extremely highly-cited publications having led a grant from one of the three bodies since January 2006 to 79 (48.2%; 95% CI: 40.5–55.8%). More specifically, 28 (17.1%) currently hold a grant as PIs from the MRC, while 37 (22.6%) have received one in the past. In addition, 27 (16.5%) are current PIs in a Wellcome Trust grant, and 29 (17.7%) have had one in the past. Finally, 16 authors (9.8%) currently lead a grant from the NIHR, while 16 (9.8%) did so in the past.

### Board members of funding bodies

When looking at the MRC board members we found that 97 out of the 188 eligible for grant members (51.6%) held an MRC grant in 2017 and 89 (47.3%) at some point in the past. In total, 123 members of the MRC (65.4%) had held an MRC grant at some stage between 2006 and 2017. In addition, 75 MRC members (40%) had had a grant from the Wellcome Trust and 19 (10.1%) from the NIHR. Overall, 148 members (78.7%) have held at least one grant from one of the three major funding bodies since 2006.

We then looked at the 168 board members of the Wellcome Trust, who were eligible to apply for a grant. When compared against the list of awarded grants, we found that 111 of them (66.1%) were grant holders of a Wellcome Trust grant in 2017 and 88 (52.4%) held a grant from the Trust in the past. That results in a total of 123 members (73.2%) who have had a Wellcome Trust grant at some point since 2006. In addition, 89 (53%) had held a grant from the MRC and 12 (7.1%) from the NIHR. Overall, a total of 147 members (87.5%) had held a grant as PIs from one of the three funding bodies at some point since 2006.

Finally, we analysed 317 members of the NIHR boards. The percentage of individuals who held a grant from the NIHR or other funding body varied depending on the Programme we were looking at. In sum, 148 members (46.7%) held a grant from the NIHR at some point since 2006 and 170 (53.6%) had a grant from any of the three major funding bodies.

**Fig 3:**
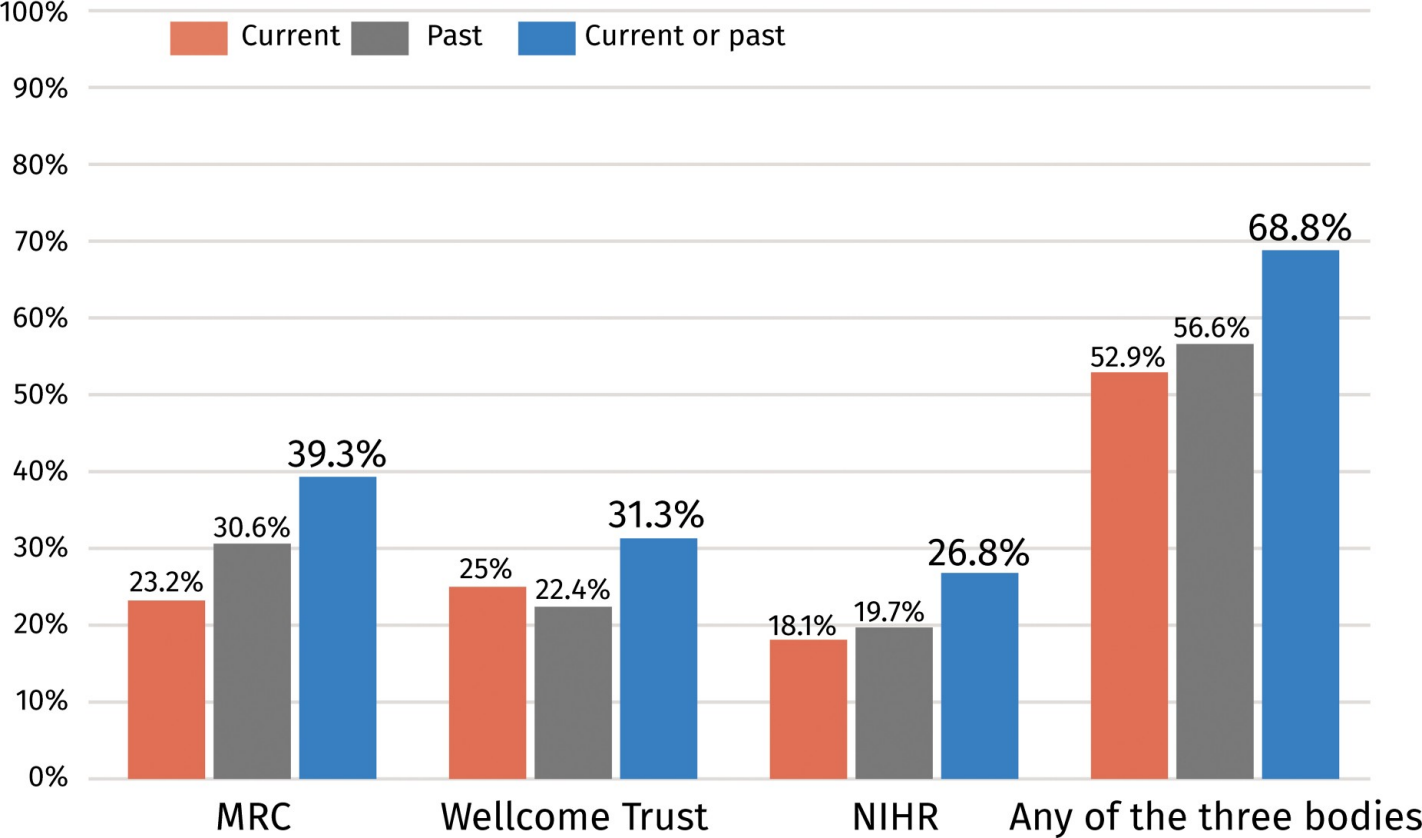
Percentage of all board members combined who have been principal investigators in a grant.

After analysing each funding body separately, we combined all board members together. Fig 3 shows the percentage of board members (664 individuals across the three bodies, after excluding duplicates) who held a grant from each and any of the three funding bodies. Our analysis shows that 261 board members (39.3%) across the three bodies had received a grant as PIs at some point since 2006 from the MRC. The equivalent number was 208 (31.3%) for the Wellcome Trust and 178 (26.8%) for the NIHR. In total, 457 board members (68.8%, 95% CI: 65.3–72.4%) across the three bodies had received a grant as PIs during the period studied.

**Fig 4:**
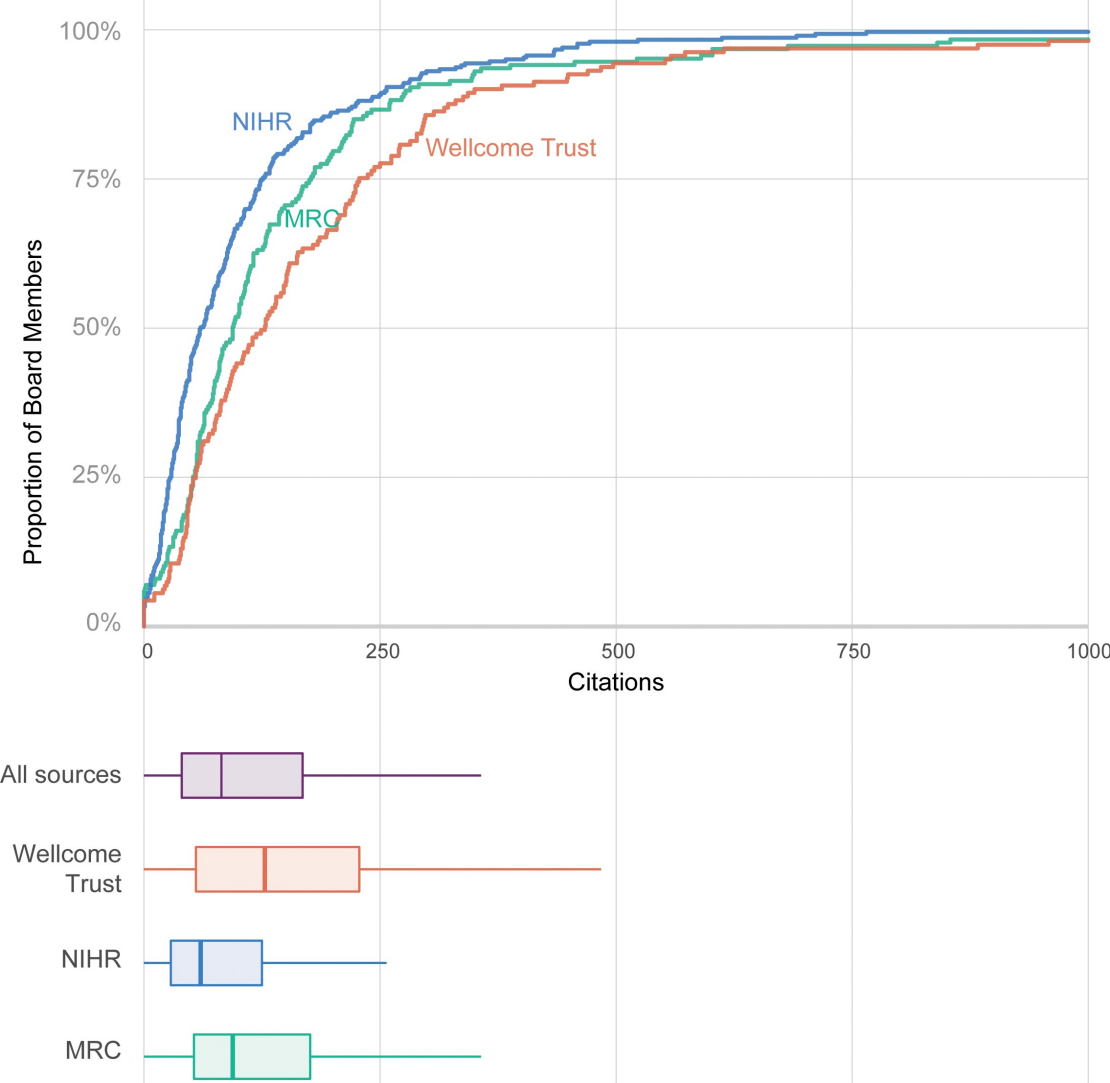
Cumulative frequency and boxplot of board members’ maximum numbers of citations to their most highly-cited paper as first, last or single authors published since 2006.

For all board members, and after removing duplicates, we identified using Scopus Application Programming Interface (API) service the most cited paper they had written as first, last or single authors from January 2006 to April 2018. From the 168 Wellcome Trust members, only 3 (1.8%) had led an extremely highly-cited paper with over 1000 citations during this period. For the MRC, the picture was quite similar: 3 out of the 188 board members (1.6%) had an extremely highly-cited paper in the period examined. Finally, from the 309 members of the NIHR that we could track down only 1 (0.3%) had led a paper with more than 1000 citations since 2006. Overall, the median (IQR) number of citations for their most cited paper was 126 (53 to 228) for the Wellcome Trust, 94 (53 to 144) for the MRC and 59 (26 to 124) for the NIHR board members (Fig 4).

## Discussion

Our study provides evidence that the majority of the most influential UK health scientists do not receive funding from the country’s three main public and charity funders. Only 36.2% of these authors have currently an active grant as PIs from the MRC, the Wellcome Trust or the NIHR. The results are comparable to the findings of Nicholson and Ioannidis [5], who showed that only 39.7% of extremely highly-cited authors in the US held an active grant from the NIH in 2012. Of course, one needs to be cautious when comparing the UK with the USA, as there are large differences in terms of the pool of academics and the funding available. In 2013, the NIH’s total health research expenditure was $26 billion where the UK’s MRC, NIHR and Wellcome Trust together spent a little more than $2.7 billion, about one-tenth of the NIH investment.[1] Yet, the picture seems to be consistent in both cases: many of the researchers who publish the most influential papers in health research may be left out of public and charity funding.

Contrasted with the picture of the highly-cited authors, we show that over two-thirds of the members of boards in the three main UK funding bodies receive funding from the same body they serve. It is expected, and to some extent desirable, that a number of board members have experience from having held a grant in the past and understand the scope and aim of the body they serve.[7] It may well also be that scientists serving on these boards are ranked highly by their peers. However, the finding is potentially alarming in that in all three cases the majority or even the vast majority of the board members held a grant as PIs from the respective funder, which could suggest that decisions on who will receive a grant may be influenced by the “money-follows-money” rather than “money-follow-excellence” principle.

Some limitations of our work need to be discussed. First, there can be debate on which types of research should be funded by public funders and charities, as opposed to other stakeholders, notably the industry. The large majority of influential randomized controlled trials are funded by the industry,[9] but this practice may not be optimal.[10] In the previous analysis of highly-cited authors and their lack of funding by NIH [5], the NIH retorted that randomized controlled trials and other evidence-based medicine tools such as meta-analyses and guidelines are not within their funding remit.[11] Indeed, the number of registered clinical trials supported by NIH declined from 1580 in 2005 to 930 in 2015 and 90% of them are quite small (<500 participants) to study hard clinical endpoints that matter.[12] NIH has also abandoned participation in influential guidelines, e.g. the National Heart Lung and Blood Institute is no longer involved in cardiology guidelines and these have been sadly left to the professional societies.[13] Such a stance means that the major funding agency for health research in the US has decided to abandon almost all health research that directly shapes health decisions and outcomes.[14] This spurious (if not plain cynical) viewpoint that evidence-based medicine should be abandoned by public funders is hopefully less tenable in the UK where the importance of evidence-based medicine is more widely appreciated. For example, guidelines in the UK are still mostly produced by the National Institute for Clinical Excellence, which is a public organization.

Second, our analysis of extremely highly-cited papers focuses on a tiny part of the far tail of citations. The vast majority of influential, important, and worthy scientific work will not attract that much attention. However, the sample of papers (and their authors) that we included is unquestionably extremely influential.

Third, we did not judge whether these extremely influential papers have been subsequently questioned as to their validity. Refutation can occur even among the most-cited papers.[15] However, it would be unlikely that refutation would be so thorough and so fast that the main authors of these papers would not be able to secure some public or charity funding for their otherwise extremely influential work because of this reason.

Fourth, citation farms may also game citation metrics and generate spuriously-highly-cited authors through generalized self-citation [16] and impossible hyper-prolific publication patterns.[17] Gaming is the most difficult to achieve for the extremely highly-cited papers that we analysed. Exceptions can occur, nevertheless, e.g. when very large teams of co-authors salami-slice numerous publications from their work and these papers get cross-cited. For example, a 2002 paper from the European Prospective Investigation into Cancer and Nutrition [18] has been cited 1052 times in Scopus as of November 2018, but at least 847 (81%) of these citations are self-citations from its multiple authors who salami-slice their work in hundreds of mostly low-quality, unreliable publications of nutritional associations.

Lastly, we acknowledge that our study is descriptive and focuses on the UK only. Therefore, our results are of limited generalisability. It would be useful to explore these patterns also in other countries and to understand the potential drivers that may account for country-to-country differences, if any.

Acknowledging these caveats, the presented results become particularly relevant in an era where there is extra pressure for research institutions and universities to generate income via public and charity funding sources. Income generation has become a vital component for promotions and career development, leading individuals fighting over grants, not over accelerating innovative ideas.[19–20] What is more, it puts significant pressure to researchers, who spend a lot of time in writing proposals as well as reviewing them and sitting on committees to judge them.[21–23] Our results seem to suggest that despite this enormous investment the system seems to function poorly when it comes to assigning the resources to the top researchers. Interventions need to be carefully studied in terms of their impact. For example, rewarding scientists with monetary bonuses based on the impact factors of the journals where they publish is widely used in some countries such as China.[24] Such practices are highly questionable,[25] because a small minority of papers published in these journals attract the largest share of citations. Other citation indicators also may be easy to game. Focusing on extremely highly-cited papers, as we did in our analysis, avoids these problems. Of course, many other aspects and dimensions of scientific, scholarly, and public/societal contribution and merit would need to be accounted.

Further work is needed to identify the mechanisms through which funding decisions are made as well as the most important factors that affect funding allocation. This work should also focus on who are the players who make the decisions. Identifying who are the best scientists is not easy, but our data suggest that probably there is plenty of room for improvement.

It is now more than a decade since the Cooksey report [3] warned funders and research institutions that the “UK is at risk of failing to reap the full economic, health and social benefits that the UK’s public investment in health research should generate” (p.17). If only the minority of the country’s most influential researchers receive funding from the major funding bodies, there is serious concern we still have not reached this investment’s full potential.

## Appendix

Scopus code:

LANGUAGE ( english ) AND PUBYEAR > 2005 AND ( LIMIT-TO ( SRCTYPE , "j " ) ) AND ( LIMIT-TO (DOCTYPE , "ar " ) ) AND ( LIMIT-TO ( SUBJAREA , "MEDI" ) OR LIMIT-TO ( SUBJAREA , "BIOC" ) OR LIMIT-TO (SUBJAREA , "PHAR" ) OR LIMIT-TO ( SUBJAREA , "IMMU" ) OR LIMIT-TO ( SUBJAREA , "NEUR" ) OR LIMIT-TO ( SUBJAREA , "NURS" ) OR LIMIT-TO ( SUBJAREA , "HEAL" ) OR LIMIT-TO ( SUBJAREA , "DENT" ) )
